# Safety and efficacy of transurethral laser therapy for bladder cancer: a systematic review and meta-analysis

**DOI:** 10.1186/1477-7819-12-301

**Published:** 2014-09-25

**Authors:** Yunjin Bai, Li Liu, Haichao Yuan, Jinhong Li, Yin Tang, Chunxiao Pu, Ping Han

**Affiliations:** Department of Urology, West China Hospital, Sichuan University, Guoxue Xiang 37, Chengdu, Sichuan 610041 China; Department of Pediatrics, West China Second University Hospital, Sichuan University, Renminnan Road 20, Chengdu, Sichuan 610041 China

**Keywords:** Bladder tumor, Laser surgery, Meta-analysis, Systematic review

## Abstract

**Background:**

Transurethral laser therapy techniques are increasingly being used in the management of bladder tumors. It has reportedly been associated with good outcomes in small case series. The objective of the present study was to review the published literature and compare transurethral laser therapy for non–muscle-invasive bladder cancer (NMIBC) and conventional transurethral resection of bladder tumor (TURBT).

**Methods:**

We performed a systematic review and meta-analysis based on randomized controlled trials (RCTs) and controlled clinical trials (CCTs) to assess the two techniques. The eligible RCTs and CCTs were identified in the following electronic databases: PubMed, the Cochrane Central Register of Controlled Trials and Embase.

**Results:**

Seven studies were included in this systematic review. The baseline characteristics of these studies are comparable. We found no statistical difference between the two techniques regarding operative time. The intra- and postoperative complications showed that the laser procedure was better than TURBT for NMIBC, including obturator nerve reflex, bladder perforation, bladder irrigation rate, duration of catheterization and length of hospital stay. In addition, the 2-year recurrence-free survival improved in the laser group than in the TURBT group.

**Conclusions:**

Our systematic review and meta-analysis suggests that laser techniques are feasible, safe, effective procedures that provide an alternative treatment for patients with NMIBC. Given that some limitations cannot be overcome, well-designed RCTs are needed to confirm our findings.

## Background

Bladder cancer is one of the most common malignant diseases of the urinary tract system. According to the American Cancer Society, an estimated 74,690 new cases of bladder cancer and 15,580 bladder cancer–related deaths occurred in 2014 [1]. Approximately 75% patients with bladder cancer present with non-muscle-invasive bladder cancer (NMIBC; formerly known as superficial bladder cancer) that is confined to either the mucosa (stage Ta, carcinoma *in situ*) or submucosa (stage T1) [2].

The current standard treatment for NMIBC is transurethral resection of bladder tumor (TURBT), followed by adjuvant intravesical chemotherapy or immunotherapy [2]. The goals of TURBT are to eradicate all visible tumors, prevent tumor recurrence and prevent progression to invasive or metastatic disease. However, when the lesions are located in the lateral bladder wall or around the ureteral orifice, optimal penetration is difficult to control during TURBT, and complications such as bleeding, bladder perforation and hydronephrosis can occur. In addition, these procedures are more likely stimulate the adjacent obturator nerve, which is in close proximity to the lateral bladder wall. This stimulation causes obturator nerve reflex (ONR), leading to inadvertent bleeding and bladder perforation [3].

New technologies, such as bipolar plasmakinetics and laser surgery, have emerged to avoid these problems and improve the efficacy of TURBT. Laser is widely used in urologic surgery and has been proved to be safe, effective and minimally invasive for NMIBC [4]. Examples of lasers include the following: neodymium, yttrium aluminum garnet (Nd:YAG), potassium titanyl phosphate (also known as green-light laser), holmium YAG (Ho:YAG) and a 2-μm continuous-wave laser (thulium:YAG (Tm:YAG) laser). Of these, holmium and the 2-μm laser TURBT are the most frequently applied treatments for NMIBC, and these treatments result in satisfactory outcomes [5]. However, because of insufficient well-documented evidence to date, it remains unknown whether laser TURBT is an effective and safe alternative to TURBT for NMIBC.

In recent years, several studies directly comparing transurethral laser therapy and TURBT have been published in an attempt to explore this issue. Although the outcomes of NMIBC after transurethral laser treatment were reported to be similar to those after TURBT in terms of oncologic and perioperative outcomes, these remain a matter of debate because results have been restricted to small sample sizes and obtained from a single research center. In addition, most studies have been nonrandomized controlled trials (NRCTs). NRCTs comparing laser treatment for bladder tumors as well as TURBT could either underestimate or exaggerate any true differences between the two procedures. However, the results of a systematic review and meta-analysis of well-designed NRCTs during surgical procedures were proven feasible and exactly similar to those of contemporaneous randomized controlled trials (RCTs) [6, 7]. Consequently, we performed a systematic review of NRCTs using meta-analysis to determine whether there were any differences between the intraoperative and postoperative outcomes in addition to oncologic outcomes between these two approaches and determine whether transurethral laser treatment techniques can be an appropriate alternatives to TURBT.

## Methods

This study doesn’t involve human subjects and does not require Institutional Review Board review or consent. In March 2014, PubMed, the Cochrane Central Register of Controlled Trials (CENTRAL) (via Ovid) and EMBASE (via Ovid) were searched using the following terms: “urinary bladder neoplasm”, “transitional cell carcinoma”, “bladder cancer”, “bladder tumor”, “urinary bladder cancer” and “laser”. The article language was restricted to English.

Two authors separately evaluated all the potentially eligible studies without prior consideration of the results and assessed the methodological quality. For a study to be considered eligible, it had to meet the following criteria: (1) The study was a RCT or a controlled clinical trial (CCT); (2) the primary NMIBC had to be pathologically confirmed; (3) the treatment intervention was transurethral laser therapy (excluding Nd:YAG laser due to its not commonly being used in bladder cancer and having several drawbacks, such as deep-tissue penetration that limits its use on filmy wall areas, particularly on the posterior side and dome of the bladder) of the bladder tumor versus conventional TURBT; (4) original data for dichotomous and continuous variables had to be provided or calculated from the data source; and (5) measures of objective and/or subjective outcomes had to be clearly defined. Studies were excluded if they met the following criteria: (1) The study was not a RCT or CCT; (2) the study was an animal study; (3) patients were diagnosed with recurrent bladder cancer, muscle-invasive bladder cancer, metastatic disease and/or upper urinary tract tumors; (4) patients had previous TURBT or transurethral resection of the prostate; and (5) there was combined use of laser and TURBT.

The methodological quality of the included studies in our meta-analysis was assessed in accordance with the Cochrane Handbook [8]. We evaluated the quality of these individual studies using the Downs and Black quality assessment method, in which a list of 27 criteria are used to evaluate both RCTs and NRCTs [9]. This quality assessment scale assesses study reports, external validity and internal validity and has been ranked in the top six quality assessment scales suitable for use in systematic reviews [10]. A higher score was associated with higher study quality. The Downs and Black score ranges were grouped into the following four quality levels: excellent (26 to 28), good (20 to 25), fair (15 to 19) and poor (<14). At the same time, a grading system (GRADE) [8] was used to assess the quality of each study included in our meta-analysis, which was based on the following five factors: (1) no limitations of the study design, (2) consistency of the results, (3) directness of evidence, (4) sufficient data and (5) minimal potential publication bias. The overall quality of a systematic review was considered to be high if multiple included studies with a low risk of bias provided consistent results regarding outcome. The quality of evidence was downgraded by one level if one of the above-mentioned factors was not met. Similarly, if two or three factors were not met, the level of evidence was downgraded by two or three levels, respectively. Therefore, the GRADE approach resulted in four levels of quality of evidence: high (A), moderate (B), low (C) and very low (D).

Data extraction was independently performed by two authors and was then cross checked. Any disagreement between the extracting authors was resolved by consensus of all authors. The primary outcome measures included ONR rate, bladder perforation rate, and recurrence-free survival, whereas the secondary outcomes included operative time, bladder irrigation rate, and the duration of catheterization and length of hospital stay. In addition to the abovementioned outcomes, additional data, including the authors’ names, publication year, number of patients and their age, tumor multiplicity, tumor size, and tumor stage/grade, were extracted from each included studies and recorded as baseline characteristics.

Statistical analysis was conducted using RevMan5.2.5. The risk ratio (RR) was used for dichotomous variables, and mean difference (MD) was used for continuous data, both with 95% confidence intervals (CI). Methodological heterogeneity was assessed during selection, and statistical heterogeneity was measured using the χ^2^ test and *I*^2^ scores. If χ^2^ heterogeneity was reported as *P* > 0.1 and *I*^2^ < 50%, heterogeneity was considered low. A fixed-effects model was used to assess the data of included studies with minimal or no heterogeneity. In contrast, a random-effects model was applied. A *P-*value for significance was set at <0.05.

## Results

**Figure 1:**
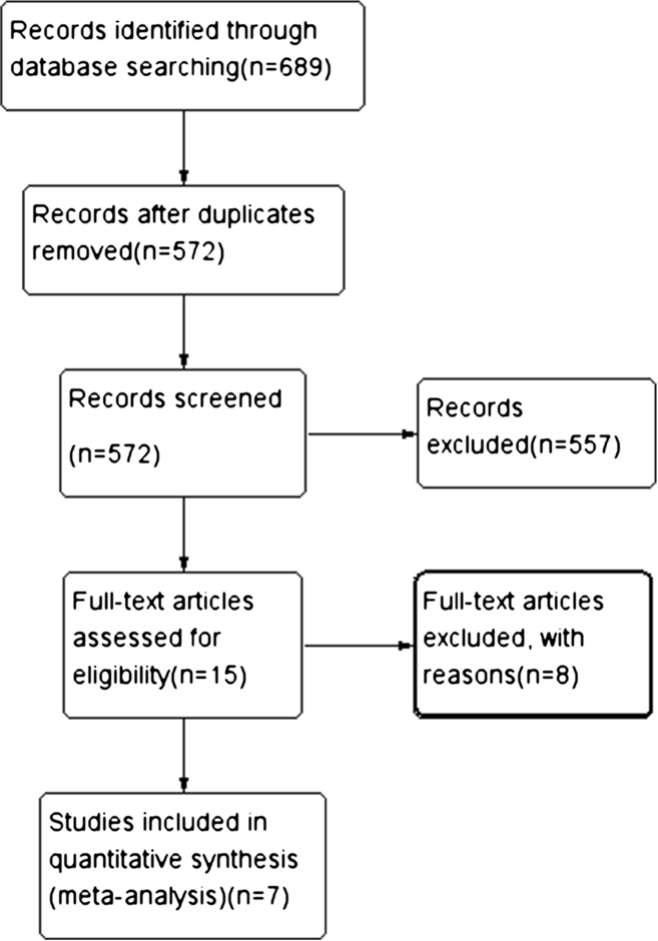
**Flow diagram of studies identified, included and excluded.**

The literature search yielded 689 reports, of which 671 were excluded on the basis of title or abstract that was irrelevant to the topic, and 8 were excluded from the remaining 15 literature after reading the full text. Therefore, data from seven studies were included in this systematic review. All of the included studies reported on various outcomes that were suitable for pooling into a meta-analysis. There were eight contrast trials, and one study included two contrast tests. Figure 1 reveals the outcomes of the literature search. The baseline and general characteristics of the included studies were extracted and are listed in Table 1. All seven study reports included the tumor size, operation time, bladder irrigation, duration of catheterization and length of hospital stay, as well as follow-up data. Five studies included reports on ONR [11–15], and six described bladder perforation [11–16]. All data from these studies that were presented as means ± standard deviation or rates, which allowed for a meta-analysis, were pooled and analyzed.

**Table 1:** **Baseline characteristics of included trials**

Trials/yr.	Designs/grade	Downs and Black score	Treatment	NO. of patients	Age (yr.)*	Male (%)	Tumor Multiplicity*	Tumor Size (cm)*	Location(n)		T Stage (n) Grade(n)					
									Lateral	Other	Ta	CIS	T1	PUNLMP	Low	High
Yang [11], 2014	Retrospective, B	18	KTP TURBT	28 32	45.3 42.5	78.6 78.1	NR NR	N/A N/A	22 17	6 15	8 7	0 0	20 25	NR NR	NR NR	NR NR
Tao [12], 2013	Retrospective, C	17	HPS	74	66.4	81.08	1.52	2.1	62	12	50	1	23	9	60	5
			TURBT	84	65.3	78.57	1.49	1.9	69	15	61	2	21	10	68	6
Liu [13], 2013	Prospective, B	20	2-micron	64	67.1	71.9	2.8	1.3	24	40	37	0	27	11	46	7
			TURBT	56	66.3	71.4	2.7	1.2	21	35	34	0	22	10	41	5
Zhong [16], 2010	Retrospective, C	16	2-micron	30	68.30	NR	1.53	2.23	NR	NR	23	2	5	4	21	5
			HoLRBT	25	65.76	NR	1.40	1.38	NR	NR	19	1	5	3	18	4
			TURBT	42	66.26	NR	1.45	1.54	NR	NR	30	4	8	7	26	9
Xishuang [14], 2010	Prospective, B	18	HoLRBT	64	72.5	81.3	2.0	1.85	25	39	36	5	23	5	39	20
			TURBT	51	74.5	78.4	1.9	1.74	20	31	30	4	17	4	33	14
Zhu [15], 2008	Prospective, B	17	HoLRBT	101	NR	78.2	NR	NR	NR	NR	67	5	34	NR	NR	NR
			TURBT	111	NR	82.9	NR	NR	NR	NR	70	7	41	NR	NR	NR
Muraro [17], 2005	Retrospective, C	16	HoLRBT	50	64.5	78	N/A	NR	NR	NR	43	0	7	NR	NR	NR
			TURBT	50	65.7	80	N/A	NR	NR	NR	46	0	4	NR	NR	NR

PUNLMP = papillary urothelial neoplasms of low malignant potential; CIS = carcinoma in situ; KTP = Potassium-titanyl-phosphate laser; HPS = 120 W high performance system Green-light laser vaporization of bladder tumor; HoLRBT = holmium laser resection of bladder tumor; TURBT = transurethral resection of bladder tumor; 2 micron = 2 micron continuous wave laser resection of bladder tumor; NR = not reported; N/A = not applicable;

* Mean or median.

**Figure 2:**
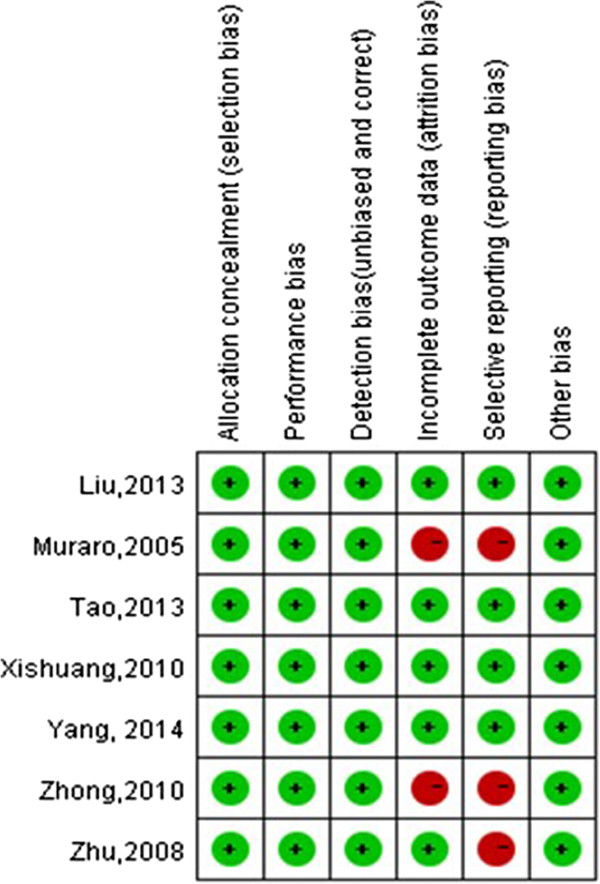
**Quality assessment. Chart summarizes our judgments about each risk of bias item for each included study.** References: Liu *et al*. [13], Muraro *et al*. [17], Tao *et al*. [12], Xishuang *et al*. [14], Yang *et al*. [11], Zhong *et al*. [16] and Zhu *et al*. [15].

Based on the Cochrane Collaboration’s tool for assessing risk of bias, the baseline characteristics of the included studies were comparable. Table 1 presents the demographics of the studies, including number of patients, age, sex, location, T stage and grade. There were no significant differences between transurethral laser resection and TURBT in any of the demographic parameters (*P* > 0.05). However, there were different levels of bias (Figure 2). Most of the studies included in our analysis were retrospective [11, 12, 16, 17], and two were RCTs [13, 15]. All included studies did not state that they were blinded, although studies on surgery can be single-blinded. The outcomes of the included studies were clear, except for those on ONR and bladder perforation [16, 17]. Overall, four studies had low risk of bias, and three [15–17] had medium risk of bias. The Downs and Black quality assessment scores of all studies were >14. The quality of results in research methodology evaluation is listed in Table 1.

**Figure 3:**
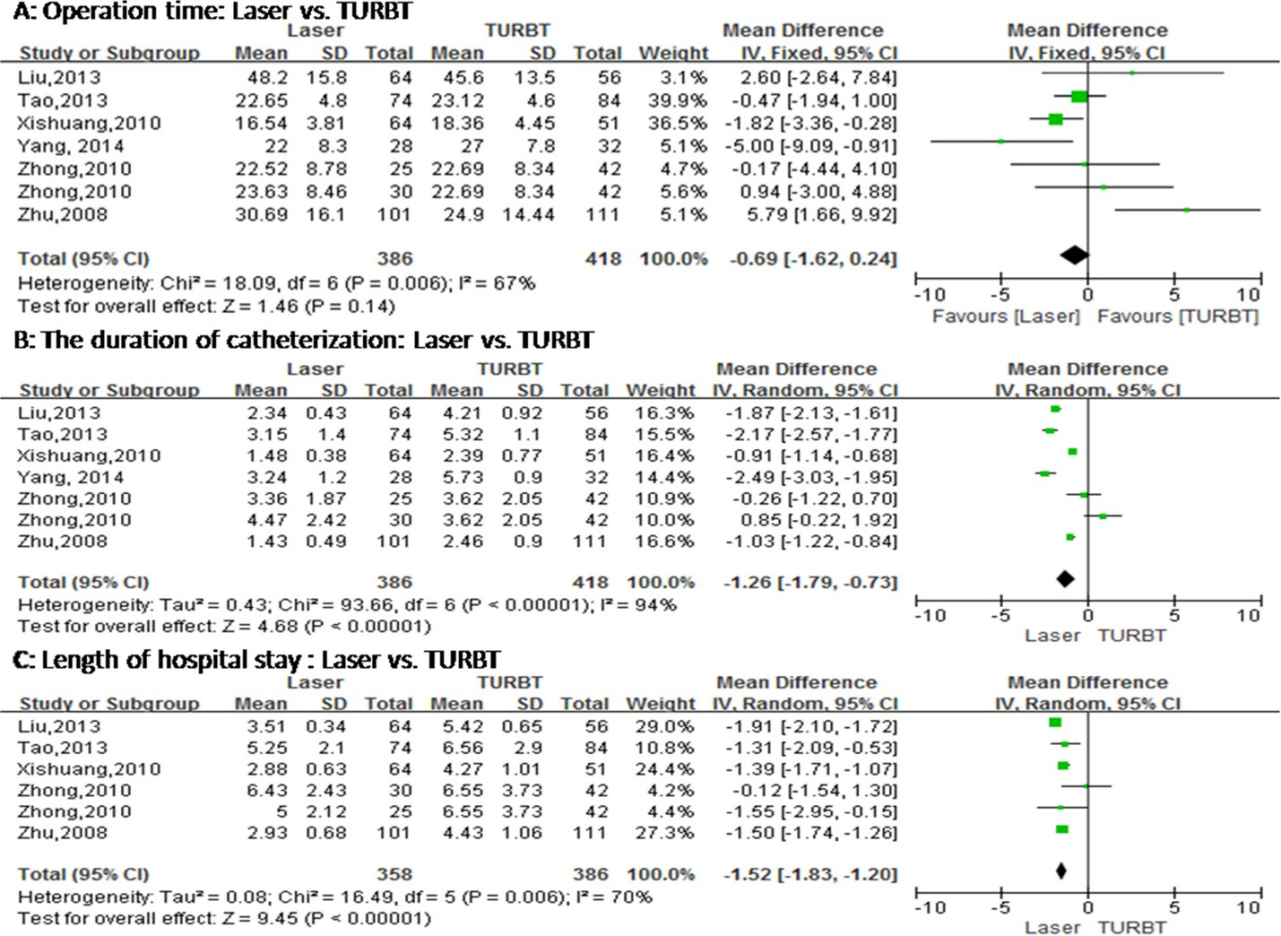
**Cumulative analysis of studies comparing laser and transurethral resection of bladder tumors with respect to perioperative clinical data. (A)** Operation time (minute). **(B)** Duration of catheterization (day). **(C)** Length of hospital stay (day). IV, Inverse variance; TURBT, Transurethral resection of bladder tumor. References: Liu *et al*. [13], Tao *et al*. [12], Xishuang *et al*. [14], Yang *et al*. [11], Zhong *et al*. [16] and Zhu *et al*. [15].

**Figure 4:**
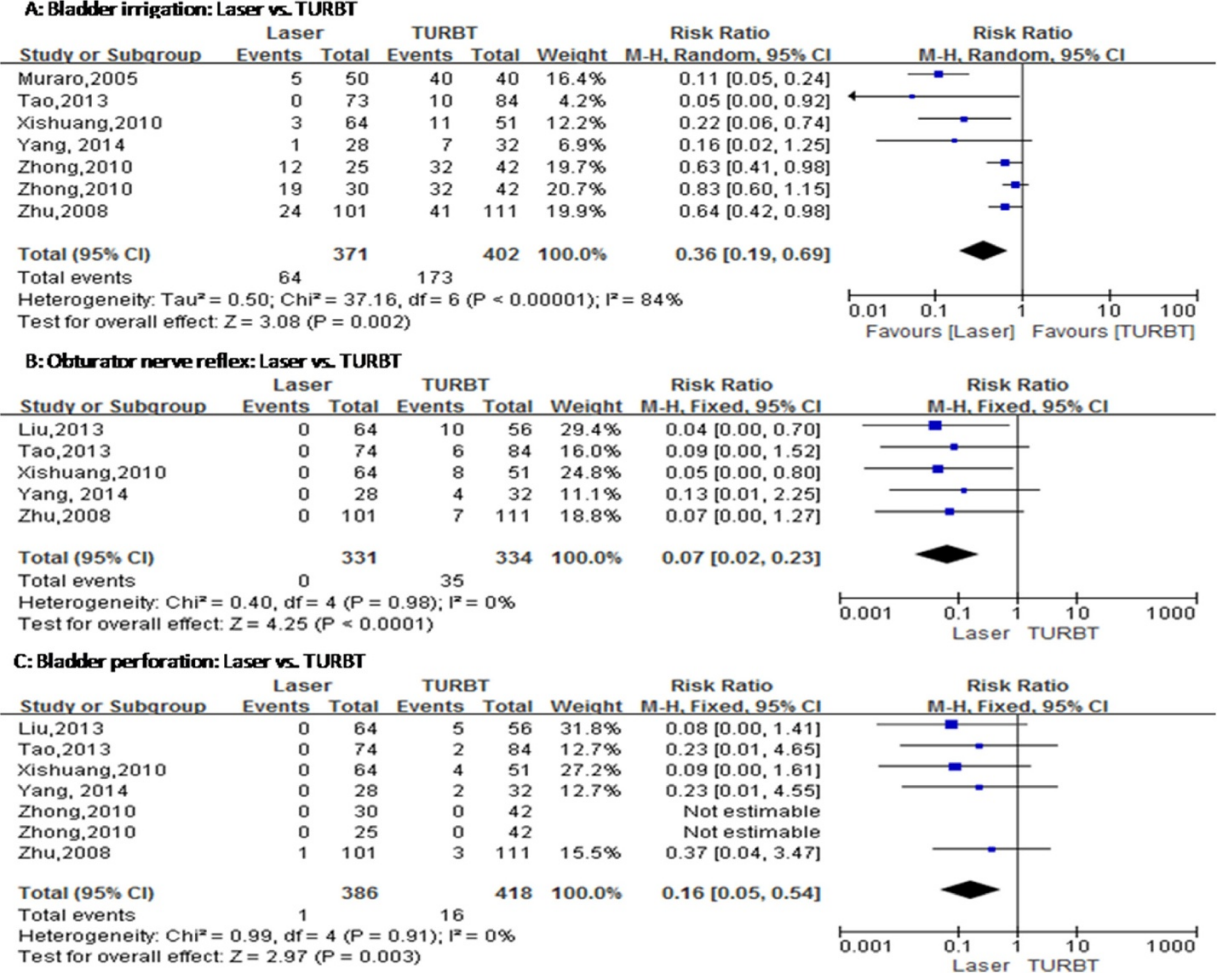
**Cumulative analysis of studies comparing laser and transurethral resection of bladder tumors with respect to intraoperative complications. (A)** Bladder irrigation. **(B)** Obturator nerve reflex. **(C)** Bladder perforation. M-H, Mantel-Haenszel; TURBT, Transurethral resection of bladder tumor. References: Liu *et al*. [13], Muraro *et al*. [17], Tao *et al*. [12], Xishuang *et al*. [14], Yang *et al*. [11], Zhong *et al*. [16] and Zhu *et al*. [15].

**Figure 5:**
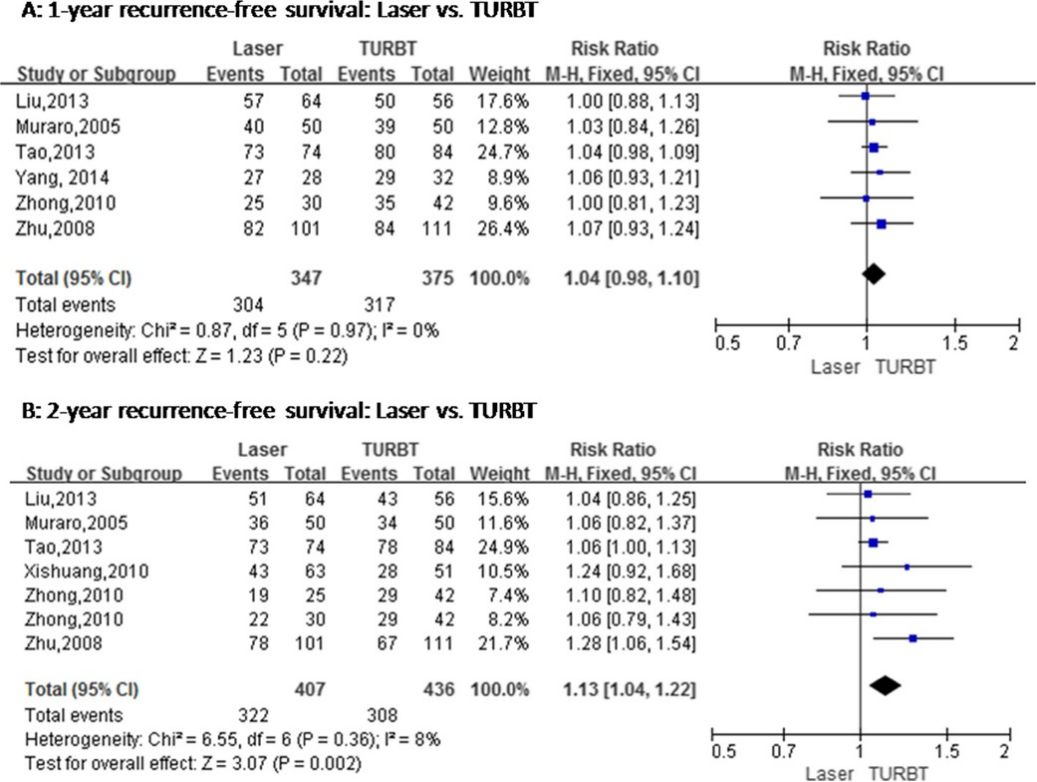
**Cumulative analysis of studies comparing laser and transurethral resection of bladder tumors with respect to recurrence-free survival. (A)** 1-year recurrence-free survival. **(B)** 2-year recurrence-free survival. M-H, Mantel-Haenszel; TURBT, Transurethral resection of bladder tumor. References: Liu *et al*. [13], Muraro *et al*. [17], Tao *et al*. [12], Xishuang *et al*. [14], Yang *et al*. [11], Zhong *et al*. [16] and Zhu *et al*. [15].

No statistical difference was found in operation time between transurethral laser treatment and TURBT for NMIBC (MD = -0.69, 95% CI [-1.62, 0.24], *P* = 0.14) (Figure 3). Six studies have compared transurethral laser management of bladder tumors with TURBT, bladder irrigation, the duration of catheterization and length of hospital stay, but these studies exhibited heterogeneity. We repeated the sensitivity analysis for these studies and obtained similar results. Using a random-effects model, the results of meta-analysis showed significant differences between the two groups with regard to bladder irrigation (RR = 0.36; 95% CI [0.19, 0.69], *P* = 0.002) (Figure 4), the duration of catheterization (MD = -1.26, 95% CI [-1.79, -0.73], *P* < 0.00001) (Figure 3) and length of hospital stay (MD = -1.52, 95% CI [-1.83, -1.20], *P* < 0.00001) (Figure 3). The two groups showed significant differences regarding ONR (RR = 0.07, 95% CI [0.02, 0.23], *P* < 0.0001) (Figure 4) and bladder perforation (RR = 0.16, 95% CI [0.05, 0.54], *P* = 0.003) (Figure 4). Although the 1-year recurrence-free survival did not statistically differ between the two groups (RR = 1.04, 95% CI [0.98, 1.10], *P* = 0.22) (Figure 5), the 2-year recurrence-free survival (RR = 1.13, 95% CI [1.04, 1.22], *P* = 0.002) (Figure 5) improved in the laser group compared to the TURBT group.

## Discussion

TURBT is commonly used in the diagnosis and treatment of primary NMIBC because it can provide adequate tissue samples for pathological examination and all visible tumors can be excised efficiently. However, TURBT is associated with potential risks, including the occurrence of ONR during surgery, especially for lesions located in the lateral bladder wall, which may lead to bladder perforation. Although the success rate of the transperineal obturator nerve block to prevent adductor muscle contractions during TURBT is 83.8% to 85.7% [18], this approach still may not completely prevent the occurrence of ONR. Fortunately, the development of bipolar plasmakinetics and the advent of modern laser therapy techniques have provided more alternatives to TURBT for bladder tumors [12, 14], which may avoid the above-listed shortcomings.

The use of laser techniques in urology was first reported by in 1978 Staehler *et al*. [19], who described the successful destruction of bladder tumors using Nd:YAG laser surgery. This laser is used mainly for the treatment of benign prostatic hyperplasia [20]. The advent of new laser techniques and improvements in conventional TURBT resulted in the abolishment of Nd lasers in the treatment of bladder tumors [5]. Therefore, in our systematic review and meta-analysis, we did not include Nd laser techniques; instead, we analyzed only commonly used lasers, such as green light, Ho:YAG and the 2-μm continuous-wave lasers.

Our review of the published data of seven comparative studies suggested that the 2-year recurrence-free survival of the laser group was better than that of the conventional TURBT group for NMIBC. Compared with conventional TURBT, laser techniques can be used to excise tumors without contact, and effective coagulation using laser vaporization can seal off the blood and lymph vessels around the tumors and reduce the implanted metastasis of tumor cells and distant tumor recurrence [21]. Furthermore, the use of laser combined with endoscopic techniques can accurately excise tumors, significantly reduce the blind areas and reduce the residual tumor. In 2001, laser techniques achieved the level of complete and precise removal of tumors from the submucosa or superficial muscle layers [22, 23]. These results were attributed to its dual functions of vaporization and resection, which provided sufficient tissue for pathological examination to assess tumor grade and stage. They also afforded a theoretical basis for follow-up treatments, except for green-light laser, because all of the tumor was vaporized. Some scholars deemed that the recurrence rate of laser treatment for bladder tumors is lower than that of TURBT because of an active immune effect of the laser techniques [23]. In contrast, an inadequate resection depth of the basal parts of tumors during TURBT to avoid bladder perforation may have resulted in a higher recurrence rate [24]. In addition, it is easier to form encrustation when using electrocautery to stop bleeding during TURBT, which may result in tumor cells remained in *situ* [25].

In our meta-analysis, intraoperative complications were less frequently observed in the laser group than in the TURBT group. Bladder perforation is the most serious complication of TURBT, and the other major causes are thermal injury and ONR during TURBT. When TURBT was applied to remove lateral tumors, the current flow passing through the obturator nerve may cause ONR, which results in sudden muscle contractions and bladder perforation. Furthermore, the temperature ranged from 100°C to 300°C at the treatment site, thereby causing thermal injury [26]. In contrast, when laser was applied to remove the tumor tissue, no current flow was produced during the procedure. This procedure could not stimulate the obturator nerve, especially in patients with NMIBC, because tumors were located in the lateral bladder wall. Therefore, bladder perforation induced by ONR can be avoided by using laser techniques. In addition, thermal injury at the treatment site is minimized, which could be attributed to the absence of a strong local electrical field.

Laser techniques without deep penetration are less invasive and therefore reduce pain perception and cause minimal bleeding. In addition, the power of the laser can be adjusted according to tumor size. The use of a laser also provides surgeons with a clearer tumor view. Furthermore, satisfactory hemostatic effects are obtained with a laser, because it can form a coagulation zone of 1- to 2-mm thickness on the surface of the wounded tissue [12]. Under these circumstances, a laser can effectively avoid additional damage due to repeated stanch, reduce bladder irrigation and shorten the catheterization time and length of hospital stay. Therefore, patients have a high degree of overall satisfaction with minimal complications.

When we pooled the included studies and assessed postoperative complications and clinical data, we found some heterogeneity. When we performed sensitivity analysis on the included studies, similar results were obtained. This may be explained by the observation that the patients were not discharged until the final postoperative pathological findings were reported [13, 15, 16]. In addition, Tao *et al*. [12] considered that most patients with a newly diagnosed bladder cancer require a hospital stay until catheter removal. However, we believe that different doctors have different standards for bladder irrigation, catheter removal and hospital discharge. Therefore, special considerations have to be incorporated when data regarding bladder irrigation, catheterization and hospitalization are pooled.

Compared with previous reviews [4, 5], our meta-analysis contained one RCT and more strict quality assessment methods were used. We evaluated the quality of these individual studies using the Downs and Black quality assessment method and also evaluated the risk of bias. Kramer *et al*. [5] published a systematic review, the results of which showed that Ho:YAG and Tm:YAG seem to offer alternatives in the treatment of bladder cancer, but there is no limit on the types of studies included and no quantitative analyses. In our review, the studies were RCTs or CCTs, and we also conducted a meta-analysis, the results of which showed that laser has several advantages over conventional TURBT. Therefore, compared with the results reported by Kramer *et al*., we deem our results more reliable. The recent review by Teng *et al*. [4], who compared only Ho:YAG laser with conventional TURBT, showed that holmium laser is safe and efficient for the treatment of NMIBC, with results similar to ours. However, we also compared green-light and Tm:YAG lasers with TURBT. Therefore, our research is more comprehensive. However, we also acknowledge that certain inherent limitations in the studies included in our meta-analysis cannot be ignored when interpreting our data. The main limitation of the present review is that only seven studies of small patient cohorts were included, and most of these were retrospective. Therefore, large-scale and multicentered RCTs should be performed to evaluate the safety and efficacy of lasers for NMIBC. Furthermore, there were differences in the length of follow-up periods, so the standard scheme and results from the long-term follow-up studies are expected. Also, none of the studies used a systematic classification system for assessing complications. In other words, there was no unified standard of bladder irrigation, catheter uproot and hospital discharge in the included studies, which resulted in heterogeneity in our pooled analysis. Nevertheless, despite varying degrees of differences in the data from the included studies, it is noteworthy that the results of each study are meaningful. In the future, the major clinical indices should have a unified standard, because only in this way will studies become more comparable, resulting in more convincing pooled analyses.

## Conclusions

Based on the data included in our meta-analysis, transurethral laser resection techniques offer improved safety and tolerability as well as enhanced recurrence-free survival compared with TURBT for NMIBC. Therefore, laser techniques are feasible, safe and effective procedures that provide an alternative treatment for patients with NMIBC. Larger, prospective, multicentered studies with a longer follow-up period should be performed to reinforce the present findings.

## Authors’ information

Yunjin Bai and Li Liu are considered as co-first authors.

## Abbreviations

CCT: Controlled clinical trial
CI: Confidence interval
NMIBC: Non-muscle-invasive bladder cancer
ONR: Obturator nerve reflex
OR: Odds ratio
RCT: Randomized controlled trial
RR: Relative risk
TURBT: Transurethral resection of the bladder tumor.
